# The plastic cell: mechanical deformation of cells and tissues

**DOI:** 10.1098/rsob.210006

**Published:** 2021-02-03

**Authors:** Kelly Molnar, Michel Labouesse

**Affiliations:** Sorbonne Université, Institut de Biologie Paris-Seine (IBPS), CNRS UMR7622, 9 Quai St-Bernard, 75005 Paris, France

**Keywords:** plastic, cell, mechanical, deformations, cells, tissues

## Abstract

Epithelial cells possess the ability to change their shape in response to mechanical stress by remodelling their junctions and their cytoskeleton. This property lies at the heart of tissue morphogenesis in embryos. A key feature of embryonic cell shape changes is that they result from repeated mechanical inputs that make them partially irreversible at each step. Past work on cell rheology has rarely addressed how changes can become irreversible in a complex tissue. Here, we review new and exciting findings dissecting some of the physical principles and molecular mechanisms accounting for irreversible cell shape changes. We discuss concepts of mechanical ratchets and tension thresholds required to induce permanent cell deformations akin to mechanical plasticity. Work in different systems has highlighted the importance of actin remodelling and of E-cadherin endocytosis. We also list some novel experimental approaches to fine-tune mechanical tension, using optogenetics, magnetic beads or stretching of suspended epithelial tissues. Finally, we discuss some mathematical models that have been used to describe the quantitative aspects of accounting for mechanical cell plasticity and offer perspectives on this rapidly evolving field.

## Graphical abstract

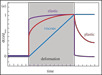

## 1. Introduction

It has long been known that cells will change their shape under the influence of mechanical stimuli [1,2]. In most cases, cells regain their initial shape after deformation. For instance, under normal physiological conditions, some cells are repeatedly stretched, as in the lung for a few seconds, or in the bladder and the intestine for several minutes, but do not remain stretched [3,4]. Likewise, cells transiently deform to properly divide, or to migrate through small pores [5]. In other cases, however, cell shape changes become irreversible during the morphogenetic processes required to generate tissues and organs during development [6–8].

Whereas *in vitro* studies have nicely outlined the physical principles guiding the cell response to mechanical forces [9,10], fewer studies have examined how embryonic cells respond to repeated deformations [11–13]. Likewise, how cells relax after large forces are removed after loading has not been characterized in as much physical detail [14].

One difference between early cell mechanics studies, which generally relied on single cells [9,10], and more recent studies involving embryos, is that the latter assemble adherens junctions that modify their mechanical properties. Another potential difference is that embryonic tissues are softer than well-differentiated cells and are supported by less complex ECM matrices [15–17]. A defining feature of most embryonic cell shape changes, and the focus of this review, is that they occur through repeated cycles of contraction and relaxation, which trigger irreversible cell shape changes over the course of morphogenesis. The irreversibility raises both physical and biological questions: how is mechanical work dissipated in living systems, and what are the molecular mechanisms driving these changes?

In this review, we discuss how cell shape change irreversibility can be attributed to mechanical plasticity, as revealed by several recent studies, and what it entails in physical terms. In particular, we compare the lessons gained from *in vivo* situations and from *in vitro* mimics of permanent deformations observed in embryos, with a focus on what we currently know about the underlying molecular causes of mechanical plasticity in cells and tissues. We mention novel experimental techniques used to study mechanical plasticity *in vitro* and present in a simple way different mathematical models derived from classical physics used to describe permanent deformations arising in tissues as a result of deformation (box 1).

Box 1. **Glossary of physical terms.**

**Active matter.** A collection of interacting self-driven particles that consume energy, resulting in collective behaviours. Examples include a flock of birds flying in a swarm, or the cytoskeleton with its component monomers assembling or Cisassembling.

**Catch bond.** A type of non-covalent interaction in which tensile force makes the bond stronger. An illustration of this principle is found in a popular child's toy, the so-called Chinese finger trap.

**Compliance.** Compliance is the inverse of stiffness. It is a measure of how an object deforms when subjected to a force (m N^−1^ in SI units).

**Dashpot.** A mechanical device often used to model purely viscous materials. A real-world example of this device can be found on the back of doors, preventing them from slamming shut.

**Elasticity.** Elasticity, generally a characteristic of solids, is the ability by which an object regains its initial shape after being deformed.

**Fluidity.** The tendency of a material to flow under a defined mechanical load rate (Pa^−1^ s^−1^ in SI units).

**Kelvin–Voigt material.** A material with both viscous and plastic properties, modelled using a spring and a dashpot in parallel. Under a constant stress, it displays no instantaneous deformation and reaches a finite stationary deformation.

**Maxwell material.** A material with both viscous and plastic and properties, modelled using a spring and a dashpot in series. Under a constant stress, it displays an instantaneous deformation and flows like a perfect fluid at long timescale.

**Figure 1.**
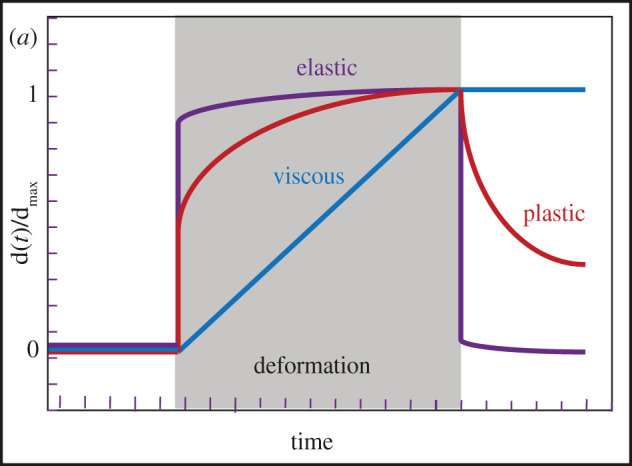
The relative deformations of elastic, plastic and viscous materials. The duration of the external stress is shown in grey.

**Plasticity.** Upon stress, a solid object will undergo a permanent deformation that will remain even when the stress is removed, unlike in the case of elasticity (figure 1).

**Power law.** A function, which has no characteristic timescale, is linear on a log–log plot. Function in the form of x*^α^*.

**Pre-stress.** Refers to the sum of all forces per unit cross-sectional area in the direction perpendicular to that area, but unlike stress it occurs before the deformation.

**Ratchet.** A stepwise and irreversible process only proceeding in one direction, examples being the common tool known as a ratchet, or a waterwheel. In the context of biology, the energy driving the motion should be provided by thermal fluctuations or by ATP hydrolysis.

**Rheology.** Field of study concerning how material flows when subjected to forces.

**Scale invariance.** When an element of an object remains the same when scales or variables are multiplied by a common factor. For example, the inner angles of an equilateral triangle are always the same, regardless of the size of the triangle.

**Solid versus liquid.** This distinction is non-trivial and actually a matter of the timescale. Some materials, usually considered as solids, might slowly flow like liquids over the span of years (glaciers for instance). A solid is a material in which the constituent atoms are firmly bonded to their neighbours and do not change position, unless a very strong force is exerted on them. A pure solid is elastic and obeys Hooke's law. A liquid is a material in which individual particles move relative to one another, despite forming weaker and transient bonds with their neighbours. Biological systems are essentially liquids, due mostly to the fact that they represent active matter able to disassociate inner networks.

**Stiffness.** Extent to which material resist deformation under applied force, inverse of compliance, measured (N m^−1^ in SI units).

**Strain.** Relative deformation that occurs when an object is subjected to mechanical stress; it is dimensionless.

**Stress.** Force per area (Pa in SI units). In actin networks, the stress can be passive, resulting from an external force (network deformation); or active, resulting from actomyosin-driven contractions of the network itself.

**Viscoelasticity.** A material with both viscous and elastic properties. Such a material regains its initial shape after deformation, but more slowly than a purely elastic body because of the viscous dampening.

**Viscoplasticity.** Similar to viscoelastic materials except that permanent deformation also occurs.

**Viscosity.** Viscosity is the degree to which a liquid resists flowing under an external stress. Viscous materials do not return to their initial shape after deformation (Pa × s in SI units).

**Young's modulus.** Ratio of stress to strain during linear elastic deformation (SI unit in Pa).

## 2. Physical parameters of plastic versus viscoelastic deformations

In materials science, an elastic solid (for instance a spring) regains its initial undeformed shape once the mechanical load is removed, whereas a viscous fluid remains deformed. Many polymers exhibit intermediate mechanical behaviours between the elastic solid and the viscous fluid, which is referred to as viscoelasticity. For large and long stresses exceeding certain thresholds, most materials enter the plastic regime in which they become irreversibly deformed. Under such conditions, molecular bonds get broken and new ones may reform. When such materials also display viscous properties, they are referred to as viscoplastic materials.

Live cells represent specific classes of viscoelastic and viscoplastic material because their mechanical architecture is more complex, because they are out of equilibrium, and because they consume energy to adapt to their environment. It is well documented that cells sense modifications to force fields in matters of seconds and then adapt to them through different mechanisms over longer timescales [18,19]. Adaptation mechanisms can involve short-term processes through mechanotransduction, leading to protein turnover, protein conformational changes or subcellular relocalization, and long-term feedbacks through gene expression. Upon deformation, cells generally exhibit a predominantly elastic behaviour at short time scales (seconds to minutes), but a mostly viscous behaviour at longer time scales [14]. In these biphasic situations, a power law often describes one of the regimes, while an exponential function represents the other [20]. Recent studies with *Drosophila* embryos suggest that the cell cortex is mostly elastic, whereas the cytoplasm is viscous [21]. In this background, the mechanical plasticity of a cell or a tissue would correspond to a permanent and irreversible deformation occurring through a change in the organization of its cortex and of the protein complexes engaged in junctions.

Classical methods to investigate the rheology of cells and tissues consist of subjecting them to a mechanical load, for instance by stretching them or deforming them locally, and then observing their recovery once the external source of deformation has been removed. Such methods have established that single-cell rheology is scale free and that cells are pre-stressed [9,10]. Depending on their geometry, their material properties, on how much cells are actively pre-stressed by molecular motors (see box 1 for a definition of pre-stress), cells stiffen or soften under mechanical stress [22–27]. In particular, following a constant stretch, cells generally stiffen, whereas cells subjected to transient stretching tend to become more fluid-like [22,28–31]. Similar observations are now being made in embryos [32]. Cell and tissue material properties largely reflect the organization of their actin cytoskeleton with their cross-linking proteins, the activity of motor proteins and the properties of the surrounding extracellular matrix [33].

Past work performed for different cell types and using different experimental methods has established that cell shape deformation often follows (sometimes only in part) a power law dependence on the time variable (see box 1) [34–36].

2.1 $$\;d(t)=c_{o}\Delta F{\left(\frac{t}{t_{0}}\right)}^{\beta },$$

where *c*_0_ refers to the compliance (see box 1) and Δ*F* is the force applied to the cell during *t_0_*. Derivatives of this equation are discussed at the end of this review. Past work performed for different cell types and using different experimental methods has established that cell shape deformation very often follows a power law dependent on the time variable, whether it is a cell restoring its shape after having borne a load or a tissue relaxing after having been stretched [34–36].

In summary, cell plasticity represents a permanent deformation of cells and tissues. As for other materials, it only occurs when a stress exceeds some magnitude or duration threshold. It frequently involves cytoskeleton or junction modifications, as will be further discussed below. In addition, mechanotransduction events can contribute to the permanent deformation by inducing signalling cascade, bringing up or facilitating cytoskeleton or junction remodelling (see also below).

## 3. Irreversible cell shape changes through ratchets

**Figure 2.**
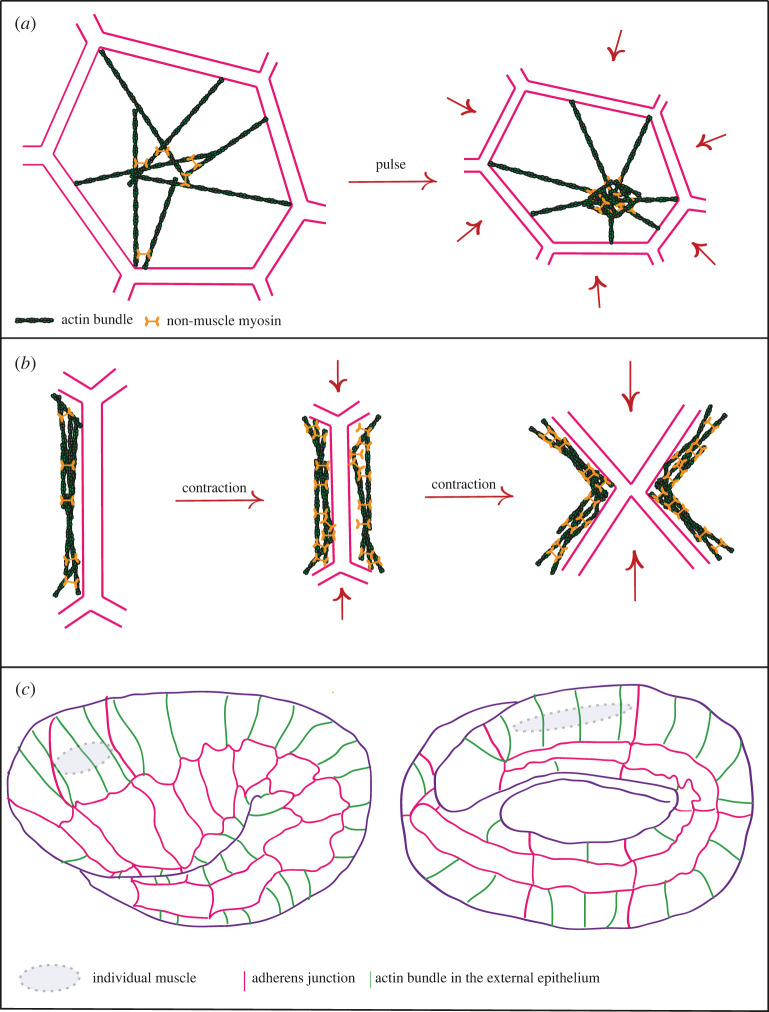
Actin cables and cell junctions. (*a*) Actomyosin pulse in *Drosophila* resulting in a cell contraction; actin in green, myosin in orange. (*b*) Role of myosin during contractions, which concentrates locally along junctions. (*c*) Second muscle-dependent phase of *C. elegans* morphogenesis, with epithelial cells (pink) and the actin cables (green); notice also a muscle cell indicated in grey (there are four longitudinal rows of ten cells, only one is shown).

Development is overall irreversible, whether at the cell fate level or at the mechanical level. A characteristic feature of many embryonic cell shape changes revealed by time-lapse studies is that they occur in a stepwise manner. Such events were first reported in *Drosophila* during germband extension through cell intercalation due to asymmetric junction shortening, or during gastrulation, dorsal closure or the formation of some placodes, which depend on apical constriction [37–40]. Similar events also take place in other species, implying their evolutionary conservation [41–45]. Past work has established that the cycles of contraction and relaxation are due to the transient formation of cortical actomyosin foci, which constrict the apical area and pull on junctions, or flow in a planar-polarized process to dorsoventrally oriented junctions and shorten them (figure 2*a,b*) [38,39,46–48].

The *Drosophila* morphogenetic events mentioned above mainly involve large and almost flat tissues. By contrast, the *C. elegans* embryo at the end of gastrulation and cell divisions can be modelled as an elongating tube. Its morphogenesis involves a different process, which nevertheless includes repeated mechanical contractions. After the first phase of elongation during which the embryo doubles its length under the influence of non-pulsatile NMYII-generated forces and of circumferentially oriented actin bundles acting as a molecular corset, the embryo depends on muscle contractions to elongate further [49] (figure 2*c*). Muscles on each side of the embryo contract alternately every 30–40 s, which locally causes tension on the dorsal and ventral epidermal cells in contact with muscles and triggers a series of biochemical reactions in those cells through a mechanotransduction process [50,51].

Given the periodicity mentioned above for fly non-muscle myosin II pulses and nematode muscle contractions, which is in a range compatible with an elastic response of epithelial cells after transient deformations, a fascinating issue is what is occurring at the molecular level when cells become permanently deformed.

Such irreversible changes have been proposed to result from a ratchet process (see box 1) [39] and thus correspond to what physicists refer to mechanical plasticity. Likewise, *C. elegans* elongation is thought to occur through a ratchet-like process because muscle contractions promote a stepwise, rather than continuous, modification of the actin corset [50]. In physics, the Brownian ratchet first introduced by Marian Smoluchowski and later popularized by Richard Feynman is a theoretical ratchet and pawl machine able to extract useful work from random fluctuations. As Feynman showed, it should not work because it would violate the second law of thermodynamics [52]. In a biological context, however, a ratchet extracting order and work from random fluctuations is possible, for two main reasons. First, biological objects consume energy—for instance biological motors consume ATP that induce oriented conformational changes. Second, many biological entities are asymmetric or polarized—for instance actin on which myosins walk has a polarity (for an in-depth discussion of ratchets in biology, see [53]). The random aspect of biological ratchets is linked to thermal noise and the fact that actomyosin pulses, or worm muscle contractions, are not equal in time and space.

Recently, two groups collaborated to design a method in tissue culture cells mimicking the ratchet-like remodelling observed in *Drosophila* during germband extension [54,55]. By genetically modifying RhoA signalling using optogenetics (see box 2), the investigators could locally and repeatedly promote actomyosin recruitment. This approach allowed them to validate a model of permanent junction deformation akin to a single-junction ratchet. Its advantage is the ability to control the duration and intensity of the contraction on junctions through laser exposure. In addition, they observed that the relative length of the junction reduced until around 20 min of applied stress, beyond which point a saturation shortening of 25% was reached.

Box 2. **Common and cutting-edge experimental techniques.**

Several recent reviews present different techniques (micropipette aspiration, parallel plate compression, FRET biosensors, laser ablation, optical/magnetic tweezers and traction force microscopy) for quantifying forces in cells, stresses *in situ* and specifically in embryonic tissues [61–63]. These reviews are easy to navigate and very complete. Therefore, the objective of this box is simply to highlight a few novel experimental techniques that are perhaps less well known: a recent advance in optogenetics, the injection of movable particles into living systems and a device for stretching suspended epithelial monolayers.

**Figure 3.**
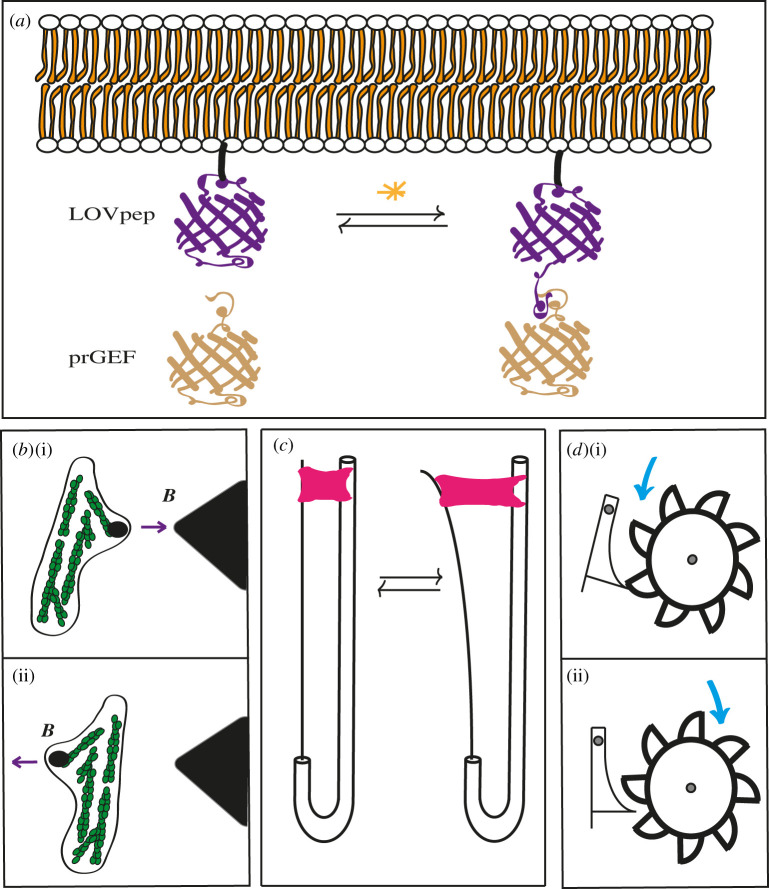
A few novel experimental techniques and ratchet. (*a*) An illustration of the LOVpep optogenetic contraction control system partially embedded in the plasma membrane. (*b*) A fibroblast containing a magnetic bead moved back and forth by the large magnet in black. (*c*) A suspended epithelial monolayer devoid of any ECM (pink) being stretched by a system of rods, the movable one also behaving as a force transducer to measure tension. (*d*) Principle of a ratchet illustrated using a waterwheel, only able to turn stepwise in one direction (i) and blocked in the other (ii).

**Optogenetics.** Optogenetic control of the RhoA GTPase signalling activity can be used with high temporal and spatial resolution [64,65], to mimic pulsatile events [54,55]. By inserting the light-sensitive protein LOVpep into cell membranes via genetic manipulation, one can use a laser to promote a LOVpep conformational change and subsequently attract a modified version of the RhoGEF LARG to the membrane. In turn, this will locally activate the RhoA small GTPase, then the Rho-kinase ROCK and the formin Diaphanous, to finally promote myosin II activation and actin polymerization [54,55]. Repeating the laser pulses several times can mimic the pulsatile events observed during fly germband extension, although through a longer pulsatile period, (figure 3*a*).

**Magnetic particles*.*** The introduction of magnetic particles and ferrofluids into living cells has been done either through injection into live *Drosophila* embryos or through phagocytosis of particles left in their environment [21,57–59]. Pushing and pulling on beads via an oscillating magnetic field (*B*) allows for measurement of mechanical properties of cells (figure 3*b*). A related approach has been to use optical tweezers to directly apply force on junctions in *Drosophila* embryos, which estimated the tension to be in the 44 pN range at the beginning of germband extension increasing to 100 pN over time [66,67].

**Monolayer stretcher.** After growing an epithelial monolayer and then stripping it from its extracellular matrix through enzymatic digestion, one can obtain a monolayer suspended in the air by two rods. The left-hand rod, which can be connected to a motorized stretching device, acts as both the stretcher and the force transducer, sensing the tension in the tissue exerted in the apparatus [20,56]. After the stretching has occurred, the tissue will dissipate the tension via lengthening (figure 3*c*).

The progressive and irreversible changes described above involve a pulse-wave force, resulting in periodic relaxation phases. Different approaches have examined the rheology of cells after stress release. In one set-up, an epithelial monolayer is grown between two parallel rods, one of which is a bendable force transducer, then denuded of its ECM. Subsequently, the monolayer is transiently stretched by at least 30% and allowed to relax [20,56] (see box 2). The relaxation curve appears biphasic, with an initial rapid elastic restoration and a slower movement towards a plastic plateau. The first phase can be recapitulated with a power law, and the second with an exponential. In addition to providing an accurate mathematical description, the distinction between the two relaxations was validated experimentally when ATP was found to be necessary for the second, but not the first phase [20]. In other set-ups, cells or embryos exposed to a magnetic field after the introduction of microbeads, ferromagnetic fluid or ferromagnetic oil microdroplets showed a fast initial partial recovery followed by a slower incomplete relaxation due to plastic changes, as described for the suspended monolayer [21,57–59]. Importantly, the cortex relaxation time is in the same range as events observed in embryonic processes such as fly gastrulation, germband extension and dorsal closure, the latter involving contraction cycles of a few minutes [21,40,47,48,60].

In summary, biological plasticity consists of irreversible deformations, which often occur in a stepwise manner. This ratchet-like behaviour is often the result of periodic force inputs, like the pulses of NMYII, or muscle contractions, meaning that they are ATP-driven as well.

## 4. Molecular causes of plasticity

Past work has highlighted the important role of actin remodelling in influencing the rheology of isolated cells [9,10,68]. Embryos present the added complexity of harbouring junctions between epithelial cells, which contribute to their mechanical properties and get remodelled upon a mechanical challenge. Hence, junction remodelling is likely to also contribute to the rheology of tissues. Recent work with different systems has indeed highlighted the crucial role of actin and junction remodelling. In addition, it has revealed the importance of stress thresholds.

### 4.1. Actin

Several recent results outline the key contribution of actin dynamics to plasticity. First, F-actin depolymerization alters the slow relaxation response of a stretched suspended monolayer, or membrane recoil after pulling on *Drosophila* embryos injected with a ferromagnetic fluid [21]. Furthermore, treating a suspended monolayer with a non-muscle myosin II (NMYII) inhibitor or a general formin inhibitor reduces the plastic relaxation of the monolayer, whereas Arp 2/3 is not required [20]. A potential word of caution on the conclusion that formins contribute to stress dissipation, which was based on the use of the SMIFH2 inhibitor, is that it may also inhibit myosin [69]. Interestingly, whereas monolayer resistance to stretching is nine times larger than that of cell pairs due to the presence of junctions and intermediate filaments, the relaxation of isolated cells and of the entire tissue both depend on formins and NMYII [20,56]. Likewise, experiments with magnetic microbeads have shown that adding paraformaldehyde, a non-specific cross-linking agent, reduces plasticity [57]. Although the latter experiments do not define a precise molecular target of paraformaldehyde they are compatible with the notion that chemical cross-linking prevents bond slippage or breakage. Interestingly, the behaviour and dynamics of actin networks depend on their architecture. *In vitro* investigations of actin rings found that a ring made of ordered actin filaments contracted, whereas a ring of disordered filaments did not [70]. Furthermore, some cross-linking is required for the contraction to occur, but too much stiffens the network, such that expressing the contractility of the network as a function of its connectivity (amount of cross-linkers present) displays a bell curve [70].

Second, as mentioned above, the second phase of *C. elegans* elongation requires muscles. These contractions cause the bending of actin cables within the epidermis and promote their severing via villin and gelsolin [50]. The cut and presumably slightly shortened actin cables are subsequently stabilized through a complex involving the α-spectrin SPC-1, the p21-activated kinase PAK-1 and the atypical formin FHOD-1. Interestingly, partial molecular dissection of FHOD-1 suggests that FHOD-1 acts by bundling or capping actin filaments rather than by promoting their polymerization [50].

Third, in *Drosophila*, it appears that a more stable actin network depending on the formin Frl/Fmnl is required to promote the propagation of pulsatile actomyosin forces [71]. Likewise, the maintenance of a sarcomeric-like actomyosin structure oriented perpendicular to the direction of tissue folding, which depends on RhoA and NMYII, is important for apical constriction [72–74]. On the other hand, although microtubules are required to promote apical constriction during gastrulation, they seem dispensable in other circumstances to promote plastic cell shape changes [21,37,75,76].

### 4.2. Thresholds

Several quite different approaches are highlighting that the step force must reach a certain level to observe a plastic deformation. First, the situation observed in *C. elegans* when muscle contractions cause the bending of circumferential actin cables before they get severed by villin [50] is highly reminiscent of *in vitro* observations showing that the actin-severing protein cofilin preferentially cuts filaments bent beyond an angle of 57° [77]. Hence, the bending angle, and thus the force produced by *C. elegans* muscles, might serve as a threshold below which no actin severing will occur. Second, during germband extension in *Drosophila*, the longer the pulsatile actomyosin foci, the more irreversible the junction length change becomes [67]. Further experiments using optical tweezers to pull laterally on junctions confirmed that the longer the force, the more permanently is the junction deflected [67]. Interestingly in this case, actin turnover was found to be important for stress dissipation during relaxation [67]. Third, in the optogenetic system described above to induce junction shortening (box 2), the experimenters had to activate the LOVpep system for a minimal amount of time to observe plastic deformation of the junction [54]. Thus, in the last two systems, a minimum amount of force must be exerted to observe a permanent change.

### 4.3. Endocytosis

**Figure 4.**
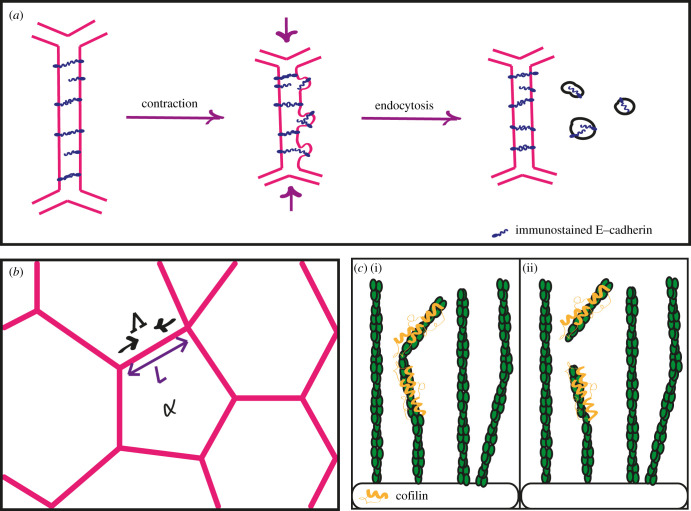
(*a*) E-cadherin along junctions is removed via endocytosis after contraction. (*b*) A typical tissue monolayer described by the vertex model, with *Λ* as the tension causing the junction *α* of length *L* to contract. (*c*) Illustration of an actin-severing protein (yellow) cutting an actin filament (green) bent beyond a certain angle *in vitro* by cofilin or in *C. elegans* by villin and gelsolin.

Several recent papers have observed a connection between changes in junction length in epithelial monolayers and the local turnover of E-cadherin. First, optogenetic control of junction contractility revealed that endocytosis of E-cadherin occurred during the shortening process. Immunofluorescence staining showed that E-cadherin first forms puncta in a formin-dependent coalescence event, then is internalized depending on dynamin [54]. These observations with culture cells recapitulate what had been observed earlier in *Drosophila* during germband extension [78]. Second, during dorsal closure, which relies on progressive amnioserosa apical cell constriction, junctions maintain their straightness despite getting shorter through the interplay between E-cadherin endocytosis and actomyosin abundance at junctions [79]. When a junction gets stretched and E-cadherin density decreases along the junction, more actomyosin flows from the medial pool to maintain junction integrity; when it gets wrinkled E-cadherin becomes endocytosed (figure 4*a*). These results point to mechanosensitive feedbacks between NMYII and junctions, as well as a regulation of endocytosis by tension. A possible connection between tension and E-cadherin tension-dependent endocytosis comes from a study showing that E-cadherin turnover is regulated by a tension-dependent relocalization of p120-catenin from junctions to the cytoplasm. As a result, E-cadherin turnover impacts on tissue viscosity [80].

In summary, permanent deformations have so far been linked to the remodelling of fibrous networks inside of cells, through either turnover or cross-linking. Actin is the most commonly reported actor in cell plasticity, and thresholds such as duration and amplitude of stress have been established. Junction plasticity has been shown to involve the traffic of E-cadherin, which is internalized during junction shortening.

## 5. Modelling cell plasticity

Models from classical physics, especially mechanics, are now the standard in biology. Here, we focus on three different ways of modelling biological plasticity. Despite including viscosity and elasticity components, it is their ability to model permanent deformations that make them unique. The approach to representing plastic deformations usually consists of an elastic model in which changing the resting length of a spring is introduced [81]. The first model considers the molecular changes occurring along junctions, the third model describes the bulk properties of the system with no specific reference to the molecular nature of the changes, while the second model mixes some molecular aspects with bulk properties.

The first is a vertex model, modified for permanent deformation of cell junctions in a tissue. The second, a Kelvin–Voigt model including a ratchet-like component, describes the elongation of the *C. elegans* embryo and involves information regarding the underlying molecular causes responsible for the plasticity of the epidermal tissue. Finally, a pair of iterated equations corresponding to an extension of a common viscoelastic power law is shown. This last model contains only a few macroscopic parameters, which very accurately reproduces the permanent changes of a fibroblast after it was subjected to multiple deformation cycles using a magnetic bead.

The vertex model is a common cellular-level description used to account for the behaviour of a two-dimensional tissue of polygonal cells [82,83]. It describes cells of different sizes interacting through tension along their shared junctions (figure 4*b*). Cells commonly change shape in a coordinated manner, causing permanent tissue deformations during morphogenesis. This can involve junction length modifications and cell intercalation initiated by junction remodelling [84]. The mechanical energy of the tissue is given by [55]:

5.1 $$E=\;1\;/\;2\underset{\alpha }{\sum }K(A_{\alpha }-\;A_{0}{)}^{2}+\;1\;/\;2\underset{\alpha }{\sum }\Gamma (P_{\alpha }-P_{0}{)}^{2}.$$

The first term describes how cells resist compression, with *A_α_* the area of cell *α*, and *A_0_* the area at rest; *K* is the elasticity constant (J m^−4^)^.^ The second describes how neighbouring cells and actomyosin contractions pull on junctions and deform them. This second term results from actomyosin contractility in the cell cortex and intercellular junctions; *P* stands for the perimeters of the cells, and *Γ* is the elastic constant for contractility. The energy equation above gives the final state of the system when the tissue relaxes. Depending on the values given for the preferred area and perimeter, the tissue can flow like a viscous liquid or rebound like an elastic solid.

In classical vertex models, the interfacial tension and junction contractility remain constant [83]. However, when junctions shorten in a pulsatile fashion, tension changes such that the model cannot explain the ratchet-like shortening of the junctions and the permanent tissue deformation (for more complete explanations, see [55]). The modified mechanical energy equation for permanent deformation is as follows:

5.2 $$E=1\;/\;2\underset{\alpha }{\sum }K(A_{\alpha }-A_{0\;}{)}^{2}+1\;/\;2\underset{\alpha }{\sum }\Lambda_{ij}\varepsilon_{ij}.$$

Here, the term *Λ_ij_* corresponds to the tension along the edge *ij,* and *ε_i_*_j_ is the attendant strain, the tension being the axial pulling force and the strain being the extent of the deformation. The two are related according to *Λ_ij_ = Yε_ij_*, with *Y* as a spring constant; this equation means that the tension along junctions varies, depending on the strain. The final step towards permanent deformation is to make the strain evolve beyond a critical strain *ε_c_.* In that case, the more a junction is compressed, the faster its resting length will shrink. Simply put, if one were to compress a junction, it would remain slightly shorter after letting go due to the fact that the derivative of the resting length *λ* (figure 4*b*) is no longer zero. This model reproduces the junction behaviour, but only for one cycle. A complete ratchet-like multi-cycle model of the junction shortening requires that the resting length and the tension are both functions of the strain [55]. In addition to its predictive power, this model involves parameters at the subcellular level, such as the tension along the junctions and the strain. This model assumes that neighbouring cells in a tissue have identical mechanical properties, which is often not the case.

A second example with an adjustable resting length describes the elongation of the *C. elegans* embryo, which proceeds in a ratchet-like manner thanks to muscle contractions (see above; figure 3*d*). As mentioned, muscles cause the bending of concentric actin cables beyond a critical angle, which causes villin to sever them, a phenomenon that has been observed directly *in vitro* using cofilin (figure 4*c*) [50,77]. In that case, the evolution of the embryo length, *l*, has been described using a modified Kelvin–Voigt model with the following equation [50]:

5.3 $$\eta \frac{dl}{dt}=-k(l\text{–}\lambda )+F_{\mathrm{epidermis}}+F_{\mathrm{muscles}}.$$

The first term consists of the viscosity *η* multiplied by the rate of the change in length d*l*/d*t*, which means that the resistance to movement increases with the speed of the movement. The next term is a spring equation with *k* as its constant and *λ* as its resting length. The final terms are forces actively generated by the muscles and the epidermis. Notice that this force equation is similar to the spatial derivative of the energy equation (5.2) in the previous model. Notice that this model assumes that the viscosity and elasticity of the entire worm can be described using two constants, *η* and *k*, which is not strictly accurate.

The force from the epidermis is generated by an active component, actomyosin contractility in the lateral epidermal cells (*F*_seam_), and a passive force depending on the same concentric actin bundles stretching circumferentially. These active and passive epidermal forces are related by

5.4 $$F_{\mathrm{epidermis}}=F_{\mathrm{seam}}\alpha_{DV},$$

where *α_DV_* is the stiffness of the actin bundles, and with the condition that *α_DV_ > 0* during elongation of the embryo. This coefficient *α_DV_*, which in effect represents the integrity of the bundles, evolves with time. The integrity is poor in the case of certain mutants, thereby preventing the force between the different cell types from being transmitted [50]. The resting length changes as a function of the speed of embryonic elongation d*l*/d*t* if the deformation exceeds a critical value, and for a non-null value of *α_DV_*.

A related model is used to describe the slow relaxation phase of a suspended epithelial monolayer using a dashpot and spring in parallel, or a Maxwell model [20]. Again, writing that the time derivative of the spring's resting length is related to the strain is sufficient to predict the permanent deformation of the tissue. A very similar approach has also been used to describe the relaxation of the *Drosophila* embryo cortex [21].

The last model we present describes the rheological properties of an isolated fibroblast, in which a magnetic bead is cyclically pulled to deform the cell (figure 3*b*). The model itself is borrowed from mechanics and is simply a modified viscoelastic power law response theory relying exclusively on compliances [57]. It represents the displacement, during and after the external deformation, respectively, as follows:

5.5 $$\mathrm{forced}\;\mathrm{deformation}:\;d(t)=(c_{ve}+c_{\;pl})\Delta F{\left(\frac{t}{t_{0}}\right)}^{\beta },$$

5.6 $$\mathrm{relaxation}:\;d(t)=c_{ve}\Delta F\left\{{\left(\frac{t}{t_{0}}\right)}^{\beta }\text{–}\;{\left((t-t_{1})/t_{0}\right)}^{\beta }\right\}+c_{pl}\Delta F{\left(\frac{t_{1}}{t_{0}}\right)}^{\beta }.$$

The parameters *c_ve_* and *c_p_*_l_ are the viscoelastic and plastic compliances. The different times are as follows: *t* is time, *t_0_* the duration of the applied force *F* and *t_1_* is when the force is removed. Only three parameters are required: the viscoelastic and plastic compliances, and the exponent of the time variable *β*. With only bulk properties as parameters, this model can describe a system for which the molecular details are unknown and can reproduce six deformation cycles.

The models mentioned above require the resting length to evolve as a function of time. To some extent, they also require information about molecular-level phenomena, such as the tension along with the junction or actin integrity. In these respects, they are different from the viscoelastic power law model, which deals with compliances (which are bulk properties) and the exponent of the time variable. In other words, these different models are implementations of plasticity at different scales, such as the cell or tissue levels.

The three models highlighted in this article are only a few among some that have been established and provide an idea of the utility, the mechanics, the complexity and the variety available. The vertex model describes junctions at the cell scale and, like the viscoplastic model for the *C. elegans* embryo that follows, requires changing a resting length. Without this extra condition, the deformation would be transient. By contrast, the third model relies on a couple of iterated equations and each iteration of the set accounts for the permanent change. This latter approach is impressive in its ability to reproduce a large number of stress/relaxation events. As mentioned, the final two approaches use continuum models describing the behaviour of the bulk of their systems. For instance, the third model describing a fibroblast did not require any information regarding the subcellular components; only bulk mechanical properties such as compliance are employed. Such considerations can be weighed when choosing a model for one's system.

## 6. Summary and outlook

We have discussed how live-cell imaging and genetic studies in embryos, as well as novel methods with culture cells to study deformation with a better handle on force magnitude and duration, are converging to highlight the importance of actin remodelling to bring irreversible cell shape changes or dissipate tension. In many cases, this irreversibility involves a ratchet mechanism with threshold levels of forces to achieve permanent deformation.

While studies have so far converged on the importance of actin and E-cadherin turnover, the molecular details have not yet been fully laid out. It will be important to define whether there are shared biophysical processes common to several cell types, or if scenarios differ depending on whether the cell undergoes apical constriction or polarized junction shrinking. The molecular nature of the feedback pathways regulating junction viscoelasticity and force dissipation remains to be worked out. In particular, the role that mechanotransduction is bound to play in such processes has not been systematically explored. We generally know that adherens junction turnover is mechanosensitive [80], that NMYII enrichment at junctions is also mechanosensitive [79] or that muscle contractions induce PAK-1 activity in *C. elegans* [51], but this must be the tip of the iceberg. Further work is necessary to identify the molecular targets of the mechanical thresholds leading to viscoplasticity and the mechanical sensors responding to tension. Work in *C. elegans* has suggested that the degree of actin filament bending could correspond to a threshold and has identified several proteins involved in actin remodelling—it will be interesting to see how general this can be. Finally, the link between the parameters in the mechanical models and molecular entities is at best tentative, and it would help to connect them. The novel approaches recently designed to probe cell mechanics *in vitro* are offering exciting perspectives to complement the power of genetic analysis *in vivo*.

## Data accessibility

This article has no additional data.
